# Seeking the neural basis of neuropsychiatric symptoms in dementia: neuroimaging findings and controversies

**DOI:** 10.3389/fnagi.2025.1633309

**Published:** 2025-08-12

**Authors:** Daichi Sone, Shunichiro Shinagawa

**Affiliations:** Department of Psychiatry, Jikei University School of Medicine, Tokyo, Japan

**Keywords:** neuropsychiatric symptoms, behavioral psychological symptoms in dementia, dementia, neuroimaging, magnetic resonance imaging, positron emission tomography

## Abstract

The biological basis of neuropsychiatric symptoms (NPS) in individuals who have dementia is poorly understood, despite the significant burden on patients, caregivers, and communities. Recent neuroimaging advances have provided reliable and less-invasive methods to investigate human brains *in vivo*. However, compared to the significant progress that has been made in the fields of diagnostic values and cognitive symptoms in dementia, the neuroimaging findings of NPS are less consistent, particularly in terms of the affected brain regions. This discrepancy may be due to differences in neuroimaging modalities or analytical methods, the fact that NPS can change over time, and/or the subjective nature of NPS assessments. In this narrative review, we summarize the extant literature on neuroimaging findings of NPS in dementia. We also discuss both the controversies and potential solutions to overcome the current problems.

## 1 Introduction

The prevalence of dementia worldwide among individuals aged ≥65 years is >10%, and an even greater percentage of people are expected to develop dementia in the coming decades (Alzheimer’s Association, 2024). It is now established that dementia is no longer just a disease of cognitive dysfunction. Neuropsychiatric symptoms (NPS) in dementia, also known as behavioral psychological symptoms in dementia (BPSD), refers to a heterogeneous group of non-cognitive symptoms and behaviors that are observed in people with dementia (Cerejeira et al., 2012), and NPS can be develop from the mild cognitive impairment (MCI) stage (David et al., 2016). Approximately 90% of individuals with Alzheimer’s disease (AD) experience one or more NPS at some point over the course of their disease (Radue et al., 2019). “Neuropsychiatric symptoms” is also included in the diagnostic criteria used for other types of dementia, e.g., visual hallucination in dementia with Lewy bodies (DLB) and behavioral disinhibition in behavioral variant frontotemporal dementia (bvFTD).

The social and economic burdens of dementia are expected to increase markedly over the next few decades (GBD 2019 Dementia Forecasting Collaborators, 2022), and thus NPS is also important in terms of social issues. In fact, NPS is a significant factor in (i) the high burdens and burnout of the individuals who are caregivers for people with dementia, (ii) nursing home placements, and (iii) the greater risks of morbidity and mortality (Tampi and Jeste, 2022). Despite the negative impacts of NPS on society, the neurobiological underpinnings of NPS have not been sufficiently elucidated, and this may hinder the development of effective treatments or interventions. Neuroimaging has been utilized to diagnose and monitor various neurological disorders and to uncover the biological mechanisms that underlie brain diseases. However, neuroimaging findings of NPS are less consistent compared to significant progress in the study of cognitive symptoms in dementia. In this narrative review, we summarize the literature concerning the neuroimaging findings of NPS in dementia, and we discuss both the current controversies and potential solutions to overcome the remaining problems. Our search strategy for literature included “neuropsychiatric symptoms in dementia”, “behavioral psychological symptoms in dementia”, and “neuroimaging” in PubMed, but strict systematic criteria in line with the PRISMA guideline were not adopted because of the narrative style of our review.

## 2 Measurement of NPS

Several scales for NPS assessment were developed after the first application of the Behavioral Pathology in Alzheimer’s Disease Rating Scale (BEHAVE-AD) in the late 1980s (Reisberg et al., 1987). The Neuropsychiatric Inventory (NPI) is widely used; its initial version included 10 items (Cummings et al., 1994), and two items have been added (Cummings, 1997). The 12-item version of the NPI consists of the following items for evaluation: delusions, hallucinations, agitation, depression, anxiety, euphoria, apathy, disinhibition, irritability, aberrant motor behavior, night-time behavior disturbances, and appetite and eating abnormalities. Many other scales are also available, including the Behavioral Syndromes Scale for Dementia (BSSD) (Devanand et al., 1992) and the Consortium to Establish a Registry for Alzheimer’s Disease (CERAD)’s Behavior Rating Scale for Dementia (BRSD) (Devanand et al., 1992). There are also several scales for specific symptoms, e.g., the Geriatric Depression Scale (GDS) (Yesavage et al., 1982) for depressive symptoms, the Apathy Evaluation Scale (AES) (Marin et al., 1991) for apathy symptoms, and the Aggressive Behavior Scale (ABS) (Perlman and Hirdes, 2008) for aggressive symptoms. A systematic review published in 2014 suggested that none of these single measures is superior to the others (Gitlin et al., 2014).

These assessment scales enable a quantitative evaluation of NPS, and this has made it possible to determine correlations between NPS and neuroimaging findings, thus greatly advancing neuroimaging research. Quantitative assessments of NPS depend largely on the measurement methodology, as is the case for many other psychiatric disorders and symptoms. Although NPS scales are useful for clinical and research purposes, scales can also be a significant limitation in investigations of the neurobiological mechanisms underlying NPS. At this time, researchers can only investigate what can be measured by scales, and scales are inevitably influenced by the subjectivity of the evaluators. Another consideration in the measurement of NPS is its multidimensional structure and socio-cultural context. It is suggested that even within a single NPS domain, there are various dimensional factors (Yi et al., 2024). To enhance diagnostic precision and better align observed symptoms with their underlying neural correlates, it is also important to consider the influence of the socio-cultural context on NPS, as with various other psychiatric disorders (Yi et al., 2024; Poon et al., 2025).

## 3 Subdomains of NPS

The issues of how NPS should be classified and whether NPS should be considered an individual syndrome or syndromes are also relevant to investigations of the neural mechanisms of and treatments for NPS. The rational classification of NPS symptoms is important for the practical use of treatment algorithms in clinical practice. For example, the European Alzheimer’s Disease Consortium (EADC) recommended classifying NPS into several distinct groups that reflect the prevalence, time course, biological associations, and psychosocial factors (Robert et al., 2005).

As certain neuropsychiatric symptoms tend to co-occur with others, many studies have employed clustering or grouping strategies, such as a principal component analysis. A systematic review made available in 2022 applied a meta-analytic method for this purpose and observed a factor structure with a good model fit for the NPI-10 (Hiu et al., 2022). Generally, delusions and hallucinations, anxiety and depression, euphoria and disinhibition, and agitation and irritability tend to be incorporated into the same category, whereas apathy can be placed in a depression category or considered an independent item (Hiu et al., 2022). Whether such a research strategy using grouping factors is truly appropriate in terms of validity remains to be established.

## 4 Neuroimaging findings of NPS

Another systematic review summarized 118 studies on neuroimaging correlates of NPS in 2016 (Boublay et al., 2016) and suggested that delusions and apathy and depression symptoms were particularly associated with brain alterations in individuals with AD. The review’s authors also indicated that the brain regions that are most relevant for NPS are mainly in the frontal lobes, particularly the anterior cingulate gyrus (ACG), although temporal, parietal, insula, and subcortical structures are also involved (Boublay et al., 2016). Following this review, many more studies made continuous efforts to clarify the brain regions associated with NPS, using newer methodologies and/or multi-center approaches. Table 1 summarizes the neuroimaging research concerning the neurobiological aspects of NPS (47 studies) published since 2016.

**TABLE 1:** Neuroimaging research regarding the neurobiological aspects of neuropsychiatric symptoms (NPS) published since 2016.

Year	First author	Subjects	Imaging modalities	Main finding(s)
2016	Bensamoun	119 AD, 308 MCI, 230 CN (ADNI)	Amyloid PET (18F-florbetapir PET)	Anxiety and irritability were associated with greater amyloid deposition
2016	Makovac	58 AD	T1WI, DTI	Specific patterns of brain atrophy and disconnection in association with each NPS in AD
2017	Huey	57 AD (ADNI)	T1WI	Apathy showed the most robust associations in the PFC, PCG, and STS
2017	Kazui	98 aMCI	T1WI, perfusion SPECT	Apathy was associated with right caudate atrophy and hypoperfusion in the cortices
2017	Ng	33 preAD, 60 AR-AD, 22 CN (ADNI)	18F-FDG PET	Greater NPS predicted subsequent hypometabolism in the PCG in preclinical AD
2017	Poulin	181 AD	T1WI	Dorsolateral prefrontal atrophy was present in persistent NPS but not in fluctuating NPS
2017	Tu	32 AD, 24 SIVD	DTI	WM integrity showed associations with multi-domains of NPS in the CC, as well as domain-specific associations in other tracts
2018	Ogama	217 AD, 39 aMCI	T1WI	Periventricular WMH in the frontal lobe was associated with verbal aggressiveness
2018	Sellami	167 genetic FTD (GENFI)	T1WI	Different neuroanatomical correlates of NPS across major forms of genetic FTD
2019	Garcia-Alberca	46 AD	T1WI	Medial temporal atrophy was associated with apathy and disinhibition
2020	Boublay	53 AD, 40 CN	T1WI	Frontal lobe volume was the most powerful predictor of the progression of the NPI score
2020	Chang	159AD	rs-fMRI	Functional connectivity parameters were associated with affective or psychotic symptoms
2020	Kanemoto	22 DLB	18F-FDG PET	Sleep disturbance was associated with hypometabolism around the thalamus
2020	Misquitta	121 AD, 315 MCI, 225 CN (ADNI)	T1WI, T2WI, FLAIR	WMH and GM atrophy both contributed to NPS subdomains
2020	Serra	101 AD, 56 aMCI, 35 CN	rs-fMRI	Patients with NPS showed functional connectivity changes that were not seen in the patients without NPS or CN
2020	Tumati	26 AD, 61 MCI, 18 CN	rs-fMRI	Global or local network alterations were observed in patients with the apathy symptom
2021	Anor	138 AD, 114 MCI (NACC)	T1WI, FLAIR	WMH predicts increases in delusions, hallucinations, agitation, depression, and irritability
2021	Jaramillo-Jimenez	55 AD, 34 DLB	T1WI	Larger amygdala volume predicts lower agitation/aggression but higher depression
2021	Matuskova	79 MCI, 37 SCI	T1WI	Reduced entorhinal cortex was associated with behavioral impairment
2021	Mohamed Nour	105 AD (ADNI)	T1WI	GM in the left insula and hippocampus was associated with depression, anxiety, and apathy
2021	Nowrangi	72 MCI/dementia	T1WI	ACC was the region most commonly associated with agitation and delusions
2021	Siafarikas	133 AD, 102 MCI	T1WI	Associations between NPS and mainly frontotemporal brain structures
2021	Tissot	26 AD, 52 MCI, 143 CN (TRIAD)	tau PET (18F-MK6240 PET)	Correlations between NPS and tau deposition
2022	Ge	151 MCI (ADNI)	tau PET (18F-AV-1451 PET)	Association between tau deposition and dyscognition was observed only in the subjects with MCI with behavior symptom(s)
2023	Cannizzaro	175 AD, 367 MCI, 223 CN (ADNI)	T1WI	Affective and vegetative NPS were associated with prefrontal and temporal areas
2023	Fan	80 AD	T1WI, FLAIR	Total or occipital WMH was associated with delusions
2023	Jiang	153 AD	T1WI, ASL	Hyperperfusion in the right ATN was associated with NPS
2023	Nakase	102 AD, 54 aMCI	T1WI, perfusion SPECT	Hyperactivity may be affected by the functional relationship between GM and WM lesions
2023	Ozzoude	126 AD/MCI, 40 ALS, 52 FTD, 140 PD, 155 CVD	T1WI, proton density, T2WI, FLAIR	WMH and reduced CT may contribute to NPS
2024	El Haffaf	216 AD, 564 MCI, 660 CN (ADNI)	T1WI	Different correlation patterns were observed between hyperactive NPS and brain regions across AD, MCI, and CN
2024	Guan	345 MCI, 912 CN (NACC)	T1WI	MBI was associated with broader brain atrophy and more rapid cognitive decline
2024	Kameyama	121 AD (J-BIRD)	T1WI	Aggressive behavior was associated with frontal lobe asymmetry
2024	Kashibayashi	180 AD, 77 aMCI (J-BIRD)	Perfusion SPECT	Brain hypoperfusion was associated with care refusal in the right hippocampus and with violence in the right temporal pole
2024	Kim	66 PD-D, 291 PD-MCI, 123 PD-SCI	T2WI, 18F-FP-CIT PET	Enlarged periventricular spaces in the temporal lobe were associated with greater NPS in the patients with PD
2024	Liampas	3,165 MCI, 4,051 CN (NACC)	T1WI	Frontotemporal atrophy was associated with elation, aberrant motor behavior, appetite disorders, apathy, and disinhibition in MCI but not in CN
2024	Marin-Marin	81 aMCI	T1WI	Lower volumes of the left temporal pole were observed in depression, and lower volumes of right middle occipital gyrus were observed in agitation
2024	Pajavand	61 MCI, 35 CN	T1WI, DTI	WM connectivity loss around the limbic system was associated with NPS
2024	Rashidi-Ranjbar	74 MCI, 143 CVD, 137 PD (ONDRI)	T1WI	Apathy was cross-sectionally and longitudinally associated with the frontal-executive circuit
2024	Tachibana	52 MCI, 38 dementia	T1WI	Associations between apathy and the insular cortex and between eating disturbance and various brain regions were observed
2024	Zhao	192 AD, 178 CN (OASIS, ADNI)	rs-fMRI	The behavioral syndrome was associated with default-mode and somatomotor networks, and anxiety syndrome was associated with the visual network
2025	Kanemoto	281 AD, 68 DLB, 180 MCI (J-BIRD)	T1WI, perfusion SPECT	Psychosis, agitation, and affective disturbance were differently associated with GM or perfusion across AD, DLB, and MCI
2025	Khoury	752 AD (NACC)	T1WI	APOE4 status can affect the CT and NPS in women and men with AD differently
2025	Lu	40 AD, 40 MCI, 40 CN	T1WI, FLAIR	Patients with NPS showed greater hippocampal atrophy and higher AD Resemblance Atrophy Index values
2025	Michelutti	16 AD, 17 MCI	T1WI	The structural covariance network was associated with affective dysregulation, impulse dyscontrol, and social inappropriateness
2025	Rashidi-Ranjbar	73 MCI, 144 CVD, 132 PD (ONDRI)	rs-fMRI	Associations between NPS and functional connectivity within the DMN, ECN, and SN were observed
2025	Sone	258 AD, 56 DLB, 185 aMCI (J-BIRD), 694 CN	T1WI	Apathy was associated with older age of the brain derived from MRI
2025	Wright	28 MCI-LB, 29 PD-MCI	rs-fMRI	Altered networks were associated with mood symptoms in PD-MCI

18F-FP-CIT PET, N-3-[18F]fluoropropyl-2β-carbomethoxy-3β-4-iodophenyl nortropane; ACC, anterior cingulate cortex; AD, Alzheimer’s disease; ADNI, Alzheimer’s disease neuroimaging initiative; ALS, amyotrophic lateral sclerosis; aMCI, amnestic mild cognitive impairment; ARAD, asymptomatic at risk for Alzheimer disease; ASL, arterial spin labeling; ATN, anterior thalamic nuclei; CC, corpus callosum; CN, cognitively normal; CT, cortical thickness; CVD, cardiovascular disease; DLB, dementia with Lewy bodies; DMN, default mode network; DTI, diffusion tensor imaging; ECN, executive control network; FLAIR, fluid-attenuated inversion recovery; FTD, fronto-temporal dementia; GENFI, genetic frontotemporal initiative; GM, gray matter; J-BIRD, a Japan multicenter study: behavioral and psychological symptoms integrated research in dementia; MBI, mild behavioral impairment; MCI, mild cognitive impairment; MCI-LB, mild cognitive impairment with Lewy bodies; MRI, magnetic resonance imaging; NACC, National Alzheimer’s Coordinating Center; NPI, neuropsychiatric inventory; ONDRI, Ontario Neurodegenerative Research Initiative; PCG, posterior cingulate gyrus; PD, Parkinson’s disease; PD-D, Parkinson’s disease with dementia; PD-MCI, Parkinson’s disease with mild cognitive impairment; PD-SCI, Parkinson’s disease with subjective cognitive impairment; PFC, prefrontal cortex; rs-fMRI, resting-state functional magnetic resonance imaging; SCI, subjective cognitive impairment; SIVD, subcortical ischemic vascular dementia; SN, salience network; SPECT, single-photon emission computed tomography; STS, superior temporal sulcus; TRIAD, translational biomarkers in aging and dementia; WM, white matter; WMH, white matter hyperintensity.

Brain morphometric imaging investigations using T1-weighted magnetic resonance imaging (MRI) account for the majority of these studies (Huey et al., 2017; Poulin et al., 2017; Ogama et al., 2018; Sellami et al., 2018; Garcia-Alberca et al., 2019; Boublay et al., 2020; Jaramillo-Jimenez et al., 2021; Matuskova et al., 2021; Mohamed Nour et al., 2021; Nowrangi et al., 2021; Siafarikas et al., 2021; Cannizzaro et al., 2023; El Haffaf et al., 2024; Guan et al., 2024; Kameyama et al., 2024; Liampas et al., 2024; Marin-Marin et al., 2024; Rashidi-Ranjbar et al., 2024; Tachibana et al., 2024; Khoury et al., 2025), with many reports implicating limbic structures such as the cingulate gyrus, entorhinal cortex, hippocampus, and amygdala as brain regions associated with NPS such as depression, apathy, agitation, and delusions (Huey et al., 2017; Garcia-Alberca et al., 2019; Jaramillo-Jimenez et al., 2021; Matuskova et al., 2021; Mohamed Nour et al., 2021; Nowrangi et al., 2021). Abnormalities in the prefrontal cortex (PFC) have also been reported (Huey et al., 2017; Poulin et al., 2017; Cannizzaro et al., 2023). Given the involvement of limbic structures and the PFC in emotional regulation and executive function, these findings may have some neuroscientific validity. However, there is variability across the studies regarding more detailed brain structures and symptom domains. Two recent studies reported associations between NPS and the insular cortex (Mohamed Nour et al., 2021; Tachibana et al., 2024). The role of the insular cortex in NPS may be of interest, as the insular cortex has recently received considerable attention for its involvement in a variety of functions, including somatosensory perception, emotion processing, and decision-making.

Considering that neuropathological changes in AD begin >10 years before the appearance of symptoms (Counts et al., 2017), the mechanism of NPS might not be elucidated by brain morphological changes alone. Several research groups have thus used multimodal neuroimaging (Makovac et al., 2016; Kazui et al., 2017; Misquitta et al., 2020; Anor et al., 2021; Fan et al., 2023; Jiang et al., 2023; Nakase et al., 2023; Ozzoude et al., 2023; Kim et al., 2024; Pajavand et al., 2024; Kanemoto et al., 2025; Lu et al., 2025). Assessments of white matter hyperintensity (WMH) in particular with the use of T2-weighted and fluid-attenuated inversion recovery (FLAIR) images have been continuous (Misquitta et al., 2020; Anor et al., 2021; Fan et al., 2023; Ozzoude et al., 2023), and it has been demonstrated that both WMH and gray matter loss contribute to NPS, which would justify a research approach combining multimodality images. Studies combining T1-weighted and diffusion tensor imaging (DTI) have also been attempted (Makovac et al., 2016; Pajavand et al., 2024). DTI can non-invasively assess white matter fibers in the brain and has been widely applied to elucidate the mechanisms of NPS (Tu et al., 2017; Zhou et al., 2023).

Cerebral blood flow and glucose metabolism are also important clinical and research targets that have been used to assess brain functions in dementia research (Kazui et al., 2017; Ng et al., 2017; Kanemoto et al., 2020; Jiang et al., 2023; Nakase et al., 2023; Kashibayashi et al., 2024; Kanemoto et al., 2025). Perfusion single-photon emission computed tomography (SPECT) is a relatively common modality in brain functional studies of NPS (Kazui et al., 2017; Nakase et al., 2023; Kashibayashi et al., 2024; Kanemoto et al., 2025). For example, cortical and hippocampal hypoperfusion has been associated with apathy or care refusal (Kazui et al., 2017; Kashibayashi et al., 2024), but given the heterogeneous results across studies, it may be necessary to confirm the reproducibility of some of these findings. Arterial spin labeling (ASL), which is an MRI technique used to non-invasively assess cerebral blood flow, has also been applied to NPS research (Jiang et al., 2023). 18F-Fludeoxyglucose positron emission tomography (FDG-PET), a nuclear medicine imaging modality that can measure cerebral glucose metabolism and is an essential modality in the treatment of dementia, is also used to study NPS, including in studies showing that the presence of NPS predicts hypometabolism in the posterior cingulate gyrus (PCG) and that sleep disturbances are associated with hypometabolism around the thalamus (Ng et al., 2017; Kanemoto et al., 2020).

Molecular imaging is an emerging topic in dementia research, and it has become a pivotal modality when coupled with disease-modifying therapies (van Dyck et al., 2022). This technique, which visualizes the deposition of proteins associated with neurodegeneration (e.g., amyloid and tau proteins), has also been applied to NPS research in recent years. It has generally been observed that NPS is more likely to be seen in individuals with greater depositions of tau or amyloid (Bensamoun et al., 2016; Tissot et al., 2021; Ge et al., 2022), but the number of such studies is limited.

The implementation of analyses of brain networks has been advocated in light of the complexity of the human brain with trillions of connections and interactions among the many regions of the brain. Several research groups have performed a brain network analysis concerning NPS, based on the functional connectivity derived from resting-state functional MRI (rs-fMRI) (Chang et al., 2020; Serra et al., 2020; Tumati et al., 2020; Zhao et al., 2024; Rashidi-Ranjbar et al., 2025; Wright et al., 2025). Associations between NPS and various networks (including the default-mode network) have been reported, but these need further validation. There are also a few reports about structural networks that were observed with DTI or morphological MRI (Pajavand et al., 2024; Michelutti et al., 2025). Decreased white matter networks around the limbic system and changes in the anatomical covariance networks are reported to be associated with NPS.

The use of artificial intelligence (AI), including machine learning, in the field of medicine has become a major trend in recent years, in brain imaging studies as well as many other fields. Nevertheless, few studies have used machine learning in NPS imaging research. Neuroimaging-based brain-age prediction is an advanced machine learning application that has been applied to calculate the age of individuals’ brains, and the gap between brain age and actual age is expected to be a novel biomarker for various neuropsychiatric disorders (Sone and Beheshti, 2022). A recent investigation of the relationship between brain-age and NPS indicates that apathy is associated with abnormal brain aging (Sone et al., 2025). Considering the advantages of machine learning analyses, these AI methods are expected to be applied to future NPS research.

As can be seen in Table 1, AD and amnestic MCI have been the most common targets of NPS studies. This may be due not only to the high prevalence of these two disorders but also to the availability of multicenter public databases, including the Alzheimer’s Disease Neuroimaging Initiative (ADNI). Other studies targeted FTD, cerebrovascular disease, or cognitive dysfunctions in Lewy body disease (Sellami et al., 2018; Kanemoto et al., 2020; Jaramillo-Jimenez et al., 2021; Ozzoude et al., 2023; Kim et al., 2024; Rashidi-Ranjbar et al., 2024; Kanemoto et al., 2025; Rashidi-Ranjbar et al., 2025; Sone et al., 2025; Wright et al., 2025). Other multicenter databases have also been accessed, including the National Alzheimer’s Coordinating Center (NACC) database, Translational Biomarkers in Aging and Dementia (TRIAD), the Genetic Frontotemporal Initiative (GENFI), the Ontario Neurodegenerative Research Initiative (ONDRI), and the J-BIRD (Japan multicenter study: Behavioral and psychological symptoms Integrated Research in Dementia). Some of these databases allow public access and some do not, which may lead to a high level of evidence in the future. In contrast, the targeted or reported subdomains of NPS are highly heterogeneous (Table 1), and thus a more unified research methodology is desired in this respect.

## 5 Integration of multimodal neuroimaging findings

Given the heterogeneity of research methods and targeted participants, it may be difficult at present to establish a unified view on the neural basis of NPS. However, focusing on studies with larger samples (*N* > 100), structural MRI studies consistently report abnormalities in the frontal and temporal lobes in NPS (Poulin et al., 2017; Ogama et al., 2018; Mohamed Nour et al., 2021; Siafarikas et al., 2021; Cannizzaro et al., 2023; Kameyama et al., 2024; Liampas et al., 2024). At a subdomain level, for aggression, frontal lobe abnormalities have been consistently reported (Ogama et al., 2018; Kameyama et al., 2024), whereas for depression and anxiety, atrophy of limbic structures was reported (Mohamed Nour et al., 2021; Cannizzaro et al., 2023). An FDG-PET study has shown that NPS predicts hypometabolism in the posterior cingulate gyrus (Ng et al., 2017), which may indicate that brain structure and metabolism in NPS are associated through the limbic circuit. Regarding other types of dementia than AD, there are still limited number of studies with relatively small sample size (mostly *N* < 100). Thus, it is not clear whether we can assume a similar neural basis of NPS in DLB or FTD to that in AD.

## 6 Future directions

Neuroimaging research that applies molecular imaging, multimodal imaging, multicenter or longitudinal data, network analyses, and machine learning are expected to play further significant roles in the future. More unified research methods are also important in terms of the reproducibility and generalizability of findings, and the same goes for the evaluations of NPS and its sub-domains.

The concept of mild behavioral impairment (MBI) has been proposed as a category of psychiatric and behavioral disturbance prior to the onset of typical cognitive symptoms in dementia (Ismail et al., 2016). Indeed, an increasing number of neuroimaging studies of MBI have been conducted (Matsuoka et al., 2023), and the expansion of the concepts of NPS may provide new insights into the relationship between psychiatric symptoms and neurodegeneration. Moreover, in addition to socio-cultural contextualization (Yi et al., 2024; Poon et al., 2025), it is also important to take into account the real-world functional impact of NPS on daily lives of patients and caregivers to advance precision medicine in this field (Karttunen et al., 2011; Chen et al., 2022).

In conclusion, although much imaging research has revealed aspects of the neural mechanisms of NPS, challenges remain, particularly in terms of the heterogeneity in methodologies and results. It is hoped that the further development of research methods will benefit both the individuals affected by neuropsychiatric symptoms and society.
